# External Application of Iodide of Mercury in Syphilitic Caries

**Published:** 1844-10-19

**Authors:** 


EXTERNAL APPLICATION OF IODIDE OF MERCURY IN SYPHILITIC CARIES.

Mr. Storks, in a case of lupus, had successfully employed the tincture of iodine, as a remedy, administered both locally and internally.

Mr. Pilcher had seen several cases in which the nasal bones had become necrosed, not from common, but from specific inflammation, such as that which resulted from syphilis. This, too, had occurred when a cure had been apparently effected, and no suspicion was entertained that the nasal bones would become diseased. Gradually, however, and almost imperceptibly, the bones gave way, and were ultimately entirely removed. These cases were generally the result of syphilis; inflammation came on in the Schneiderian membrane, the bone then became affected, and not being sufficiently nourished, became gradually absorbed. He had seen the nasal bones affected in this manner as the result of scarlet fever, where that disease affected the nose. The inferior spongy bone he had often seen affected in this way. He related the case of a young lady who, after scarlet fever, suffered from inflammation of the lining membrane of the nose, which was followed by necrosis of the inferior spongy bone; on removing this she got quite well. The reason the bone perished rather than the cartilage resulted from the lower degree of organisation of the former. In these cases, however, he did not think that it was always the nasal bones which were affected; the vomer might be destroyed, or some other of the neighbouring bones. Mr. Pilcher then made some remarks on the value of the Talicotian operation, and on the eligibility of carrying out the principle, if possible, in cases of cleft palate and other congenital or acquired deformities.

Some cases were related of perforation of the cartilaginous septum of the nose resembling a hole punched out by a shoemaker's punch. It was the result of inflammation. The local application of nitric acid, however, stopped the ulceration.—*Lancet:*

